# 

**Published:** 1914-12-26

**Authors:** 


### Medical Appointments.

Mr. A. L. Leon has been appointed deputy-chairman of the Midwives Act Committee of the London County Council.

Dr. Nicholas M. Donnelly, M.B., B.S., B.A.O. (Ireland), has been appointed senior assistant medical superintendent of the St. Pancras South Infirmary, London, and Miss M. K. Bishopp, M.R.C.S., L.R.C.P., has been appointed junior assistant medical superintendent at the same institution.

Dr. Alexander Ambrose, who is Coroner for Walthamstow, has gone on service with the Territorial Force. Dr. Ambrose is a B.A., LL.B., M.D. (Dub.), M.B., B.Ch., and took his D.P.H. at Cambridge in 1899.

### The Book Market.

LIST OF NEW BOOKS RECEIVED FROM THE FOLLOWING PUBLISHERS:—

Hodder and Stoughton, for the University of London Press, Ltd.: "National Health Insurance through Approved Societies." By W. Addington Willis, LL.B. Price 10s. 6d. net.

Edward Arnold: "Surgical Materials and their Uses." By Alexander McLennan. 4s. 6d. net.

Henry Frowde and Hodder and Stoughton: "A Nursing Manual for Nurses and Nursing Orderlies." By Duncan C. L. Fitzwilliams, M.D. 6s. net.

Office of *The Sphere* and *The Tatler*: "Winter's Pie, 1914." 1s. net.

### Institutional and Public Reports.

Report for 1914: Lady Minto's Indian Nursing Association.

A Short Account of the Orphans' Home, Austral Street, West Square, London, S.E., being the Forty-eighth Annual Report. By Charlotte Sharman. Hudson and Kearns, Hatfield Street, S.E. Price 2d.

Rates of Subscription: Twelve months, 6/6; Six months, 4/-; Three months, 2/-, post free to any address in the United Kingdom. Abroad, Twelve months, 8/8; Six months, 5/-; Three months, 2/6, post free.—Address The Subscription Dept., "The Hospital," The Hospital Building, 28/29 Southampton Street, Strand, London, W.C.

